# 

**Published:** 1843-07

**Authors:** 


### THE BRITISH AND FOREIGN MEDICAL REVIEW

OR

### QUARTERLY JOURNAL

OF

### PRACTICAL MEDICINE AND SURGERY

STATE OF LOUISIANA CHARITY HOSPITAL * MEDICAL LIBRARY * JUSTICE

LIBRARY OF THE

Orleans Parish Medical Society

EDITED BY

JOHN FORBES M.D. F.R.S. F.G.S.

---

VOL. XVI.

JULY—OCTOBER 1843

LONDON

JOHN CHURCHILL PRINCES STREET SOHO

MDCCCXLIII.

### BOOKS RECEIVED FOR REVIEW.

ENGLISH.

1. Remarks on Medical Reform; in a second letter to Sir James Graham. By Sir James Clark, Bart.—London, 1843. 8vo, pp. 40. 1s.

2. Animal Magnetism. By Edwin Lee, Esq. Third Edition.—London, 1843. 8vo, pp. 86. 2s. 6d.

3. The Life of a Travelling Physician, from his first introduction to practice; including twenty years wanderings through the greater part of Europe. In Three Vols. 8vo, pp. 312, 304, 294.—Lond. 1843. 1*l.* 11s. 6d.

4. A Treatise on Mental Derangement. By Francis Willis, M.D. Second Edition, revised.—London, 1843. 8vo, pp. 136.

5. Fallacies of the Faculty, &c. By Samuel Dickson, M.D.—London, 1843. Royal 8vo, pp. 188. 2s. 6d.

6. A Lecture on Quack Medicines, delivered to the Wakefield Mechanic's Institution. By T. G. Wright, M.D.—London, 1843. 8vo. pp. 44.

7. A concise Historical Sketch of the Progress of Pharmacy in Great Britain. By Jacob Bell.-Lon. 1843. 8vo, pp. 108. 1s. 6d.

8. Guy's Hospital Reports. Second Series. No. I. April, 1843. 6s.

9. A Register of Experiments performed on Living Animals; disclosing new views of the Circulation, &c. By James Turner, Veterinary Surgeon. Part II.—London, 1843. 8vo, pp. 100.

10. An Essay on the Nature and Treatment of Apoplexy. By J. A. Gay, M.D. —Paris, 1808. Translated by E. Copeman, M.R.C.S., with an Appendix.—Lond. 1843. 8vo, pp. 94. 3s. 6d.

11. A System of Phrenology. By George Combe. Fifth Edition. Two Volumes. —Edinb. 1843. 8vo, pp. 519, 442. 21s.

12. On the Amendment of the Law of Lunacy. A Letter to Lord Brougham. By a Phrenologist.—London, 1843. 8vo, pp. 39.

13. Homœopathy, with Notes, &c. By Edwin Lee, Esq. Third Edition.—Lond. 1843. 8vo, pp. 51. 1s. 6d.

14. Bloodletting as a Remedy for the Diseases incidental to the Horse and other Animals. By Hugh Ferguson.—Dublin, 1843. 8vo, pp. 82.

15. On Spasm, Languor, Palsy, and other Disorders, termed Nervous, of the Muscular System. By J. A. Wilson, M.D. &c.—London, 1843. 8vo, pp. 201. 7s.

16. The Baths of Germany considered with reference to their remedial efficacy in Chronic Diseases. By Edwin Lee, Esq. 2d Ed.—Lond, 1843. 8vo, pp. 268. 7s. 6d.

17. On the Anatomy and Diseases of the Urinary and Sexual Organs. By G. J. Guthrie, F.R.S.—Lond. 1843. 8vo. pp. 155. Third Edition. 5s.

18. Practical Remarks on Gout, Rheumatic Fever, and Chronic Rheumatism of the Joints. By R. B. Todd, M.D., F.R.S. &c.—London, 1843. 8vo, pp. 216. 7s. 6d.

19. The Plea of Humanity and Common Sense against Two Publications—The Stammerer's Handbook and Mr. Yearsley's, &c.—London, 1843. 8vo, pp. 51.

20. General Therapeutics and Materia Medica, adapted for a Medical Text-book. By R. Dunglison, M.D. &c. In Two Vols. —Philadelphia, 1843. 8vo, pp. 515, 489.

21. A Practical Treatise on the Diseases of the Testis and of the Spermatic Cord and Scrotum. With Illustrations. By T. B. Curling, &c.–Lon. 1843. 8vo, pp. 540. 18s.

22. Austria: its Literary, Scientific, and Medical Institutions, &c. By W. R. Wilde, M.R.I.A.—Dub. 1843. 8vo, pp. 325. 9s. 6d.

23. Treatise on the Dental Art, founded on actual experience. By F. Maury. Translated from the French, with Notes and Additions. By J. B. Savier.—Philadelphia, 1843. 8vo, pp. 285.

24. A Practical Treatise on the Diseases of Women. By S. Ashwell, M.D. Part II. Organic Diseases.—Lond. 1843. 8vo. 8s.

25. A Conspectus of the Pharmacopœias, &c. By A. T. Thomson, M.D. The second American Edition. Edited by C. A. Lee, M.D.—New York, 1843. 12mo, 288.

26. The Transactions of the Provincial Medical and Surgical Association. Vol. XI.—London, 1843. 8vo, pp. 567. 21s.

27. Mental Hygiene, or an Examination of the Intellect and Passions, designed to illustrate their influence on health and the duration of life. By W. Sweetser, M.D.—New York, 1843. 8vo, pp. 270.

28. Clinical Remarks on certain diseases of the Eye, &c. By J. C. Hall, M.D.—London, 1843. 8vo, pp. 228. 7s.

29. Manual of British Botany, according to the natural orders. By C. C. Babington, M.A.—London, 1843. 8vo, pp. 400. 9s.

30. Medical History of the Expedition to the Niger during the years 1841-2, comprising an account of the fever which led to its abrupt termination. By J. O. M'William, M.D. With Plates.—London, 1843. 8vo, pp. 296. 10s.

31. Essays on Surgical Pathology and Practice. By A. Watson, M.D., F.R.C.S.E., &c. Parts I & II.—Edinburgh, 1843. 4to, pp. 70.

32. The Art of Living. By Dr. Henry Duhring.—London, 1843. 8vo, pp. 144.

FOREIGN.

1. Traité Pratique sur les Maladies des Organs genito-urinaires. Par le Docteur Civiale. 2d Edition. 1e Partie.—Paris, 1843. 8vo, pp. 588.

2. De Anatomia Pathologica Pedis equini et vari. Auctore J. C. Weis.—Arhusii, 1842. 8vo, pp. 97.

3. In Tuberculorin Pulmonum Dissertatio. Auctore Olavo Glas, M.D.—Holmiæ, 1839. 8vo, pp. 84.

4. Mémoire sur l'Emploi des Carbonate d'Ammoniaque dans la Scarlatine, &c. Par le Docteur Rieken.—Bruxelles, 1843. 8vo, pp. 120.

5. Thèse sur le Delirium Tremens ou Folie des Ivrognes. Par le Docteur Bouyard.—Bruxelles, 1843. 8vo, pp. 163.

6. Mikroscopiske Undersögelser af Nervesystemet. Ved Adolph Hannover, Lic. Med.—Kjöbenhavn, 1842. 4to, pp. 112.

7. Ueber den Generations wechsel, order die Fortpflanzung und Entwickelung, &c. Von J. J. S. Steenstrup, Lector an der Academie in Sorö.—Copenhagen, 1842. 8vo, pp. 140.

8. Den Pathologiske Anatomies Svar paa Spörgsmaalet; Hvad er Cancer? Ved Adolph Hannover, L.M.— Kjöbenhavn, 1843. 8vo, pp. 106.

9. Die Geschichte der Phrenologie. Von Gustav von Strave.—Heidelberg, 1843, 8vo, pp. 60.

